# Accelerated myocardial T1 mapping using SMART1Map and compressed sensing with temporal PCA

**DOI:** 10.1186/1532-429X-16-S1-O9

**Published:** 2014-01-16

**Authors:** Jeff A Stainsby, Li Zhang, Graham A Wright

**Affiliations:** 1 GE Healthcare, Toronto, Ontario, Canada; 2 Imaging Research, Sunnybrook Research Institute, Toronto, Ontario, Canada

## Background

Myocardial T1 mapping has been proposed for characterizing a wide range of diffuse and focal fibrotic pathologies. Due to the demands of requiring multiple sample points along the signal relaxation curve in a breath-held ECG-gated scan, current T1 mapping methods have focused on single-shot acquisitions. Collecting all data in a quiescent time window has limited T1 mapping to a reduced number of k-space lines, often with parallel imaging. These compromises lead to low spatial resolution and/or low signal-to-noise (SNR) images. This work explores the initial feasibility of accelerating the recently proposed SMART1Map method[1] using Compressed Sensing with temporal Principle Component Analysis (CS-tPCA)[2] to obtain higher spatial resolution and higher SNR T1 maps.

## Methods

A phantom consisting of 20 samples with different T1s (200-1600 ms) was imaged using SMART1Map with a matrix size of 256 × 256 over 36 cm, acquiring 6 time samples (TS) of the T1 recovery curve at 100, 315, 1315, 2315, 3315, and 4315 ms. Images were acquired with a single-channel body coil resulting in low SNR data. The fully sampled k-space data was retrospectively undersampled in the phase-encoding direction, by fully sampling the central k-space lines (25%) and discarding the outer k-space lines randomly to yield a net acceleration factor of 2. With singular value decomposition on a model-based training matrix, the orthogonal principal components (PC) of the time evolution of the signal were extracted. Both image sparsity and prior knowledge in the signal recovery dimension are incorporated in the CS framework to facilitate robust reconstruction of the PC coefficient maps from undersampled datasets. The images at each TI are then obtained by a coefficient-weighted sum of PCs. T1 maps generated using the original fully sampled data and from the CS-tPCA reconstructed data were compared.

## Results

**Figure 1:**
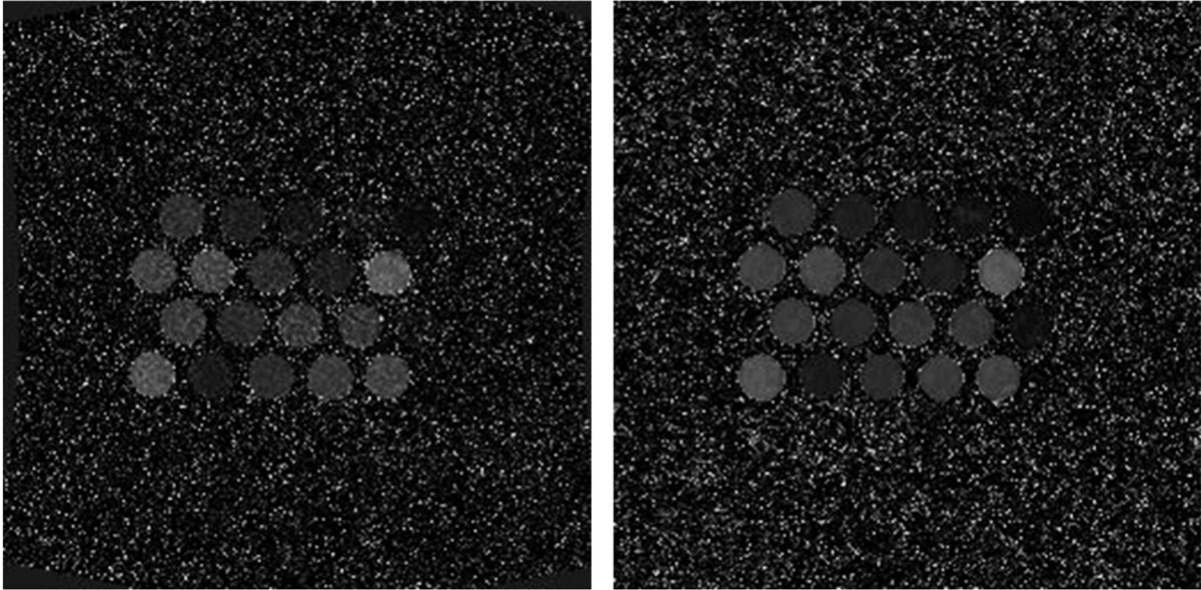
**Reconstructed T1 maps using fully sample original source data (left) and using two-fold undersampled data and the CS-tPCA reconstruction (right)**. The CS-tPCA reconstruction reduces the variability within the samples in the T1 maps, with the mean variability across all samples dropping from 39% to 12% of the sample T1.

**Figure 2:**
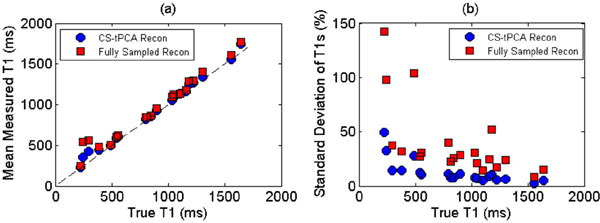
**(2) Mean measured T1 values plotted versus actual T1 values based on a gold-standard reference IR-prepared Spin Echo acquisition**. With the effective noise reduction achieved with the CS-tPCA the average T1 error dropped from 17% with the fully sampled data (red squares) to 8% with the CS-tPCA reconstructed data (blue circles). (b) Standard deviation of T1 values within each sample. T1 maps using fully sampled data (red squares) demonstrate more variability than with CS-tPCA data (blue circles).

T1 maps using the fully sampled and CS-tPCA reconstructed data are shown in Figure 1. Figure 2a plots the mean T1 values in the samples compared to the actual T1 values illustrating that the accuracy of T1 mapping was improved with CS-tPCA reconstruction (mean error across all samples of 8% vs 17%). Due to the low SNR input data, the average standard deviation of T1 values across individual samples (Figure 2b) was 39% for the original fully sampled data which dropped to 12% after CS-tPCA despite using 2 times less k-space data.

## Conclusions

Accelerating T1 mapping by at least two-fold is feasible by applying Compressed Sensing with temporal Principal Component Analysis. Furthermore the CS-tPCA enables the reconstruction of T1 maps with less variability than fully sampled data. This approach would enable corresponding increases in spatial resolution (e.g. for characterizing infarct homogeneity) or decreases in acquisition window durations (e.g. for systolic imaging of right heart disease).

## Funding

N/A
